# Tetracyclines in Rheumatoid Arthritis: Dual Anti-Inflammatory and Immunomodulatory Roles, Effectiveness, and Safety Insights

**DOI:** 10.3390/antibiotics14010065

**Published:** 2025-01-10

**Authors:** Mislav Radić, Andrej Belančić, Hana Đogaš, Marijana Vučković, Andrea Gelemanović, Andrea Faour, Ivan Vlak, Josipa Radić

**Affiliations:** 1 Department of Internal Medicine, School of Medicine, University of Split, 21000 Split, Croatia; mislavradic@gmail.com; 2 Department of Internal Medicine, Division of Rheumatology, Allergology and Clinical Immunology, Center of Excellence for Systemic Sclerosis in Croatia, University Hospital of Split, 21000 Split, Croatia; 3 Department of Basic and Clinical Pharmacology with Toxicology, Faculty of Medicine, University of Rijeka, Braće Branchetta 20, 51000 Rijeka, Croatia; 4 Department of Neurology, University Hospital of Split, 21000 Split, Croatia; hana.dogas@gmail.com; 5 Department of Internal Medicine, Division of Nephrology and Dialysis, University Hospital of Split, 21000 Split, Croatia; mavuckovic@kbsplit.hr; 6 Mediterranean Institute for Life Sciences (MedILS), University of Split, 21000 Split, Croatia; agelemanovic@medils.unist.hr; 7 Vancouver Coastal Health, Vancouver, BC V5S 1M9, Canada; andrea.faour20@imperial.ac.uk; 8 Department of Physical Medicine and Rehabilitation with Rheumatology, University Hospital Split, 21000 Split, Croatia; ivst.05@hotmail.com

**Keywords:** rheumatoid arthritis, tetracycline, minocycline, doxycycline

## Abstract

Rheumatoid arthritis (RA) is a chronic autoimmune disease characterized by persistent inflammation, joint pain, and progressive cartilage and bone erosion. Despite advancements in RA management with disease-modifying antirheumatic drugs (DMARDs) and biologics, some patients remain refractory to conventional treatments. Tetracyclines, such as minocycline and doxycycline, exhibit anti-inflammatory and immunomodulatory properties, making them potential supplementary treatments. This narrative review explores their effectiveness, mechanisms of action, safety profiles, and current challenges in RA care. Tetracyclines have demonstrated significant immunomodulatory effects, including the inhibition of pro-inflammatory cytokines and matrix metalloproteinases (MMPs), which are critical in RA pathology. Clinical trials, including double-blind, placebo-controlled studies, have shown efficacy in reducing RA symptoms, particularly in early and refractory cases. However, their use remains limited by inconsistent evidence, small sample sizes, and concerns about antimicrobial resistance. Current guidelines for RA management do not explicitly recommend tetracyclines due to these limitations, although off-label use may be considered in specific cases. The use of tetracycline for RA is restricted by drug interactions causing bacterial resistance alongside unpredictable patient responses, hence the necessity for prudence in its prescription within a clinical setting. To overcome these limitations, the development of safer compounds, in-depth in silico analyses, and integration with personalized medicine approaches are needed. Overall, tetracyclines show promise as adjunct therapies in RA management due to their dual anti-inflammatory and immunomodulatory actions. This review highlights the need for further research to address gaps in evidence, including the development of modified tetracyclines with reduced antimicrobial effects and improved safety profiles, as well as the integration of personalized medicine approaches to optimize patient outcomes.

## 1. Introduction

Rheumatoid arthritis (RA) is a chronic autoimmune disease characterized by persistent inflammation in synovium, joint pain, as well as stiffness alongside the progressive erosion of cartilage and bone, leading to significant disability when not treated adequately. Systemic manifestations such as fatigue and extra-articular involvement are common, marking its complicated clinical presentation even more [1,2,3]. The disease affects approximately 0.5–1.0% of the global population, with prevalence consistent across diverse populations, highlighting its widespread impact on public health [4].

The pharmacological management of RA follows a stepwise approach, incorporating nonsteroidal anti-inflammatory drugs (NSAIDs), glucocorticoids, disease-modifying antirheumatic drugs (DMARDs) [conventional synthetic (cs)DMARDs, biological (b)DMARDs, targeted synthetic (ts)DMARDs], and JAK inhibitors, aimed at controlling inflammation, relieving symptoms and halting disease advancement [5]. Minocycline, as well as doxycycline, are members of the tetracycline class of semi-synthetic antibiotics that possess anti-inflammatory properties and are used to treat various inflammation-related diseases, including RA [6,7,8].

Due to sulfasalazine’s combined anti-inflammatory and antibacterial qualities, antibiotics were first used to treat RA in the 1930s [9,10]. Tetracyclines gained attention in the 1960s and 1970s because of their immunomodulatory properties, as evidenced by research showing that they effectively reduced the symptoms of RA [11,12,13,14]. Recent clinical trials have examined macrolides such as clarithromycin and roxithromycin, which have demonstrated potential advantages mainly associated with their anti-inflammatory actions [15,16,17,18].

Significant anti-inflammatory and immunomodulatory effects are exhibited by tetracyclines, particularly doxycycline and minocycline, which reduce the generation of pro-inflammatory cytokines and T cell proliferation. These substances prevent the presentation of antigens, the activation of microglial cells, and the synthesis of important inflammatory chemicals like cyclooxygenase-2, phospholipase A2, and nitric oxide. Tetracyclines also efficiently block matrix metalloproteinases (MMPs), which may be used therapeutically to treat autoimmune disorders and diseases linked to inflammation, such as RA [19,20]. Concerns about scarce evidence and methodological inconsistency, followed by antimicrobial resistance, safety, and cost-effectiveness concerns, are the main reasons for the limited acceptance of tetracyclines in current clinical guidelines [21,22,23].

It is projected that 17.6 million individuals of all ages worldwide had RA in 2020, a 121% increase from 1990 [24]. The global prevalence rate increased by 14.1% from 1990. It is projected that 31.7 million people worldwide will have RA by 2050, representing an 80.2% rise in cases from 2020 to 2050, based on projected demographic trends, which highlights the significant burden of this disease and further emphasizes the significance of exploring alternative therapeutic options [24].

This narrative review aims to give an all-inclusive look at the role of tetracyclines, notably minocycline and doxycycline in RA management, focusing on their historical evolution, mechanisms of action, and supporting clinical evidence. By critically evaluating the current body of evidence and umbrella guidelines, this review discusses patient selection criteria, safety profiles, and challenges in integrating these drugs into everyday clinical practice, including issues such as drug resistance and variations in patient responses. Furthermore, this review highlights emerging research and future directions to optimize the use of tetracyclines in RA care.

## 2. Mechanism of Action of Tetracyclines in RA

Tetracyclines are a group of antibiotics that inhibit protein synthesis by preventing the binding of aminoacyl-transfer RNA to the messenger RNA-ribosome complex [10]. This is primarily achieved through binding to the 30S ribosomal subunit in the messenger RNA translation complex [10]. Their anti-inflammatory effects are attributed to the inhibition of enzymes involved in the inflammatory cascade [10].

Minocycline, one of the most extensively studied tetracyclines, has demonstrated anti-inflammatory effects in patients with RA [25]. T cells play a critical role in RA pathogenesis, and studies have shown that minocycline suppresses T cell activity by acting on the T cell receptor (TCR)/CD3 complex [25]. This leads to the reduced production of pro-inflammatory mediators, including interleukin-2 (IL-2), interferon-gamma (IFN-γ), and tumour necrosis factor-alpha (TNF-α) [25]. Minocycline also inhibits T cell proliferation by decreasing IL-2 responsiveness [25]. Further research has demonstrated that minocycline suppresses NFAT1-mediated transcriptional activation pathways in human CD4^+^ T cells [26,27]. In rat models, minocycline significantly reduced the incidence of adjuvant-induced and collagen-induced arthritis—both considered T cell-dependent models of RA due to their pathophysiology [28].

Minocycline has also been shown to suppress antigen-presenting capacity. Both minocycline and doxycycline inhibit the production of pro-inflammatory cytokines, such as TNF-α, interleukin-1 beta (IL-1β), and interleukin-6 (IL-6), as well as matrix metalloproteinases (MMPs), particularly MMP-2 and MMP-9 [19]. Additionally, tetracyclines inhibit inflammation-associated molecules, including inducible nitric oxide synthases (iNOSs), phospholipase A2 (PLA2), cyclooxygenase-2 (COX-2), and prostaglandins [19].

**Figure 1:**
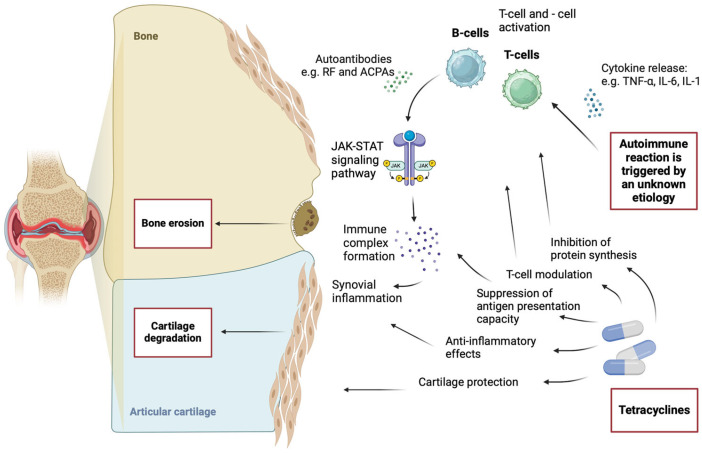
Pathogenesis and mechanism of action of tetracyclines in RA. Created in BioRender. F, A. (2024) https://BioRender.com/p56c644, (accessed on 26 December 2024).

RA and osteoarthritis (OA) progression often involve cartilage damage accompanied by the spontaneous release of nitric oxide (NO). A study evaluating the effects of tetracyclines on this process found that doxycycline and minocycline inhibited nitric oxide synthase enzymes, including OA-specific NOS and rodent inducible NOS (iNOS) [29]. The mechanism of action was determined to involve the regulation of RNA expression and enzyme translation [29]. A detailed visualization of the pathogenesis of RA and the mechanism of action of tetracyclines is presented in Figure 1.

## 3. Tetracyclines in RA: A Historical Perspective

The exploration of tetracyclines in the treatment of RA has a rich history, evolving from their initial recognition as antibiotics to their current understanding as modulators of collagenase activity, which is critical in the pathology of RA. The earliest significant findings regarding tetracyclines’ therapeutic roles emerged in the 1970s, when researchers began to examine their effects beyond antimicrobial properties. In particular, a study conducted by Rokkanen et al. [30] highlighted tetracyclines’ ability to suppress collagenase activity, implicating them in the management of conditions characterized by excessive tissue degradation, such as RA. This discovery laid the groundwork for further investigations into the biochemical mechanisms by which tetracyclines could exert protective effects on joint tissues.

Subsequent research, particularly noted in studies conducted by Skinner et al. [31] and Tourtellotte [32], explored the interaction of tetracyclines with human synovial tissues. In these studies, minocycline was administered to RA patients, showing a significant 67% reduction in collagenase activity post-treatment. This provided compelling evidence that tetracyclines could inhibit the destructive enzymatic processes associated with RA, suggesting a dual mechanism of action: antimicrobial effectiveness and the modulation of MMPs. The researchers proposed that this non-antimicrobial action could lead to the development of novel therapeutic strategies aimed at mitigating tissue damage caused by collagenases, a hallmark of RA pathology. The emergence of chemically modified tetracyclines (CMTs), such as the dedimethylaminotetracycline examined by Tourtellotte [32], represented a significant advancement. These modifications aimed to retain anti-collagenase properties while eliminating antibiotic effects, thus reducing the risk of resistance. Preliminary animal studies demonstrated that CMTs could effectively reduce collagenase activity without affecting glucose metabolism, suggesting a favourable safety profile for long-term use in chronic conditions like RA.

By the 1990s, clinical trials began to substantiate the therapeutic potential of minocycline in RA treatment. Studies conducted by Kloppenburg et al. [11] and Pruzanski et al. [33] assessed minocycline’s safety and efficacy over extended periods. In a notable trial, 40 patients with active RA received minocycline, revealing statistically significant improvements in multiple clinical parameters, including joint tenderness and swelling. The absence of severe adverse effects further underscored minocycline’s promise as a safer alternative to traditional RA therapies. A larger multicenter trial [34] confirmed these findings, demonstrating that minocycline administration led to substantial improvements in disease activity metrics after 48 weeks. Notably, patients treated with minocycline exhibited higher rates of improvement in joint swelling and tenderness compared to placebo, reinforcing the drug’s clinical relevance in managing RA symptoms.

In summary, the research trajectory on tetracyclines in RA has transitioned from their initial application as antibiotics to a nuanced understanding of their role in collagenase inhibition and tissue protection. These studies collectively underscore the potential of minocycline and its derivatives as effective therapeutic agents, warranting further exploration and validation through controlled clinical trials to establish their mechanisms of action and long-term safety in the treatment of RA. This evolving narrative highlights the importance of repurposing existing medications to address complex chronic conditions, ultimately enhancing patient care in rheumatology.

## 4. Clinical Evidence of Tetracyclines in RA Treatment

The first studies exploring the use of tetracyclines and their effectiveness in treating RA were conducted during the 1970s and 1980s. Minocycline was the most commonly studied antibiotic from the tetracycline group in these trials, which initially compared its efficacy to that of a placebo. Patients in these studies had previously been treated with DMARDs. The findings consistently demonstrated that minocycline was superior to placebo, generally safe, but with an unclear mechanism of action [11,12,13].

Two notable studies, conducted by Kloppenburg and colleagues [11] and the Rheumatoid Arthritis Investigational Network (RAIN) study group [13], had relatively small sample sizes, which was a significant limitation. In the late 1990s, the RAIN group investigated minocycline, comparing its effectiveness to hydroxychloroquine in DMARD-naive RA patients [13]. The results showed that the minocycline group achieved better outcomes, including a positive effect on glucocorticoid-sparing strategies. The drug’s efficacy was particularly pronounced in patients with more aggressive forms of RA, who made up the majority of the study population. At that time, minocycline was suggested as a DMARD with high potential for RA treatment [14].

Doxycycline, another tetracycline-group antibiotic, was also tested as a potential treatment for RA. In one study, doxycycline was compared to azithromycin and placebo, but the results showed no significant reduction in disease activity across any of the three groups [35].

Given methotrexate’s (MTX) established role as one of the most effective DMARDs for RA treatment, another study examined the combination of MTX and doxycycline versus MTX alone [36]. This study included 66 seropositive RA patients with disease durations of less than a year, none of whom had received prior DMARD therapy [36]. The primary endpoint, ACR50, was achieved more frequently in the group treated with doxycycline plus MTX, demonstrating superior outcomes [36]. However, the authors emphasized the need for further studies to understand the mechanism of action [36].

The largest observational study to date was conducted in the United States between 1998 and 2009, involving 15,716 patients with RA who were prescribed tetracyclines (minocycline or doxycycline) [37]. The study concluded that tetracyclines were primarily used in refractory cases rather than as a first-line treatment [37]. They were well tolerated, and the most common side effects were nausea, dizziness, and skin complications [37].

Additionally, the combination of oral tetracyclines with clindamycin was tested as an RA treatment [38]. However, this study was discontinued after the initial results from 20 patients failed to achieve the desired ACR50 positive response [38]. Table 1 is a summary of all listed studies.

## 5. Current Guidelines and Clinical Use of Tetracyclines in RA

Current guidelines for managing RA do not explicitly include tetracyclines as a recommended therapeutic option. The European Alliance of Associations for Rheumatology (EULAR) guidelines employ the Oxford Centre for Evidence-Based Medicine Levels of Evidence to shape recommendations [21]. The American College of Rheumatology (ACR) guidelines utilize the GRADE framework, combining systematic reviews with a voting panel of clinicians and patients to reach a consensus on the strength and direction of recommendations [22]. The National Institute for Health and Care Excellence (NICE) integrates evidence interpretation with economic considerations in its methodology [23]. Despite their differing approaches, all these guidelines focus on established DMARDs and biologics, without highlighting tetracyclines.

The limited inclusion of tetracyclines stems from inconsistent evidence, small-scale trials, and methodological heterogeneity, as well as concerns about antimicrobial resistance, safety risks, and economic factors. However, off-label use may be cautiously explored on a case-by-case basis when standard treatments fail to achieve an adequate clinical response. In particular, tetracyclines may be considered in refractory cases to leverage their immunomodulatory and anti-inflammatory properties, especially in patients with early-stage disease or coexisting conditions like infections, where other treatments pose higher risks. This approach requires careful monitoring to ensure that the benefits outweigh the potential risks.

## 6. Challenges and Limitations in Tetracycline Use for RA

Despite the benefits mentioned in this review, the use of tetracyclines in RA may carry some risks, and, in some cases, precautions should be taken with regard to antimicrobial resistance, drug interactions, or variations in response to treatment.

The global tetracycline resistance rate in European countries for methicillin-resistant Staphylococcus aureus (MRSA) and Streptococcus pneumoniae was 8.7% and 24.3%, respectively [39], and, for Escherichia coli and Klebsiella species producing extended-spectrum β-lactamase (ESBL), the percentages were 66.9% and 44.9%, respectively [40]. Tetracycline resistance occurs through active efflux, ribosomal protection, decreased permeability, target mutation, enzymatic degradation, and gene transfer via plasmids and transposons [41].

When it comes to drug interactions, tetracyclines interact with several medications and must be carefully considered in clinical use. Doxycycline may prolong the prothrombin time in patients taking warfarin, and concomitant use with penicillin is not recommended due to the impairment of bactericidal activity. The absorption of doxycycline may be reduced by antacids, iron, zinc, and other cation-containing drugs, necessitating separate dosing. Enzyme inducers such as phenobarbital and rifampicin may accelerate the metabolism of doxycycline, resulting in subtherapeutic levels, while alcohol shortens the half-life of doxycycline. Caution should be exercised when administering doxycycline concomitantly with methotrexate, ciclosporin, retinoids, or methoxyflurane due to the increased risk of toxicity, and it may inactivate oral typhoid vaccines [42]. Similar precautions should also be taken with minocycline [43]. Furthermore, in terms of dietary interactions, milk and other dairy product consumption alongside tetracycline use can interfere with drug absorption by 50 to 90% or more [44]. Considering this substantial interaction, dairy products, in general, should be avoided during tetracycline use, which can potentiate lower adherence to this therapy.

There is limited evidence regarding the differential response of patients to RA treatment with tetracyclines. They have been studied in naive RA, early RA, long-term RA, and RA unresponsive to DMARD treatment [45], but there is a gap in knowledge about the differences between specific RA groups based on disease severity and other possible contributing factors.

## 7. Future Directions and Emerging Research

Although tetracyclines pose an interesting therapeutic option for RA, due to inconsistent evidence from small-scale clinical trials and concerns about antimicrobial resistance, the future of tetracyclines as an RA treatment remains far from common usage. In order to achieve this, the research community must first address all the current limitations and explore novel therapeutic applications, such as the development of safer compounds, in-depth in silico analyses, and integration with personalized medicine approaches.

A promising area of research is the synthesis of novel chemically modified tetracyclines that retain anti-collagenase properties while eliminating antibiotic effects, thus reducing the risk of antimicrobial resistance. Molecular modelling studies and docking can predict the interactions between these newly chemically modified or synthesized tetracyclines with their target protein(s) in order to identify compounds with high binding affinity and specificity. In addition, these novel compounds should be investigated for their synergistic effects with current therapeutic options for RA, which could enhance outcomes and reduce the doses of more toxic drugs, which could add to the long-term cost-effectiveness from the macroeconomic point of view. Recently, nanotechnology has also emerged as a promising new method for targeted drug delivery, which in turn can enhance bioavailability and reduce the systemic side effects of new drugs [46,47]. Nanotechnology also offers great potential in delivering a combination of multiple drugs, which would pose a great advantage for RA treatment as tetracyclines are proposed as an adjunct therapy option.

Emerging research in this field also lies in addressing the personalized medicine approach and the use of various omics potential. Advances in genomics and pharmacogenomics offer the opportunity to identify biomarkers that predict patient responses to tetracyclines. This would, in turn, help with patients’ stratification based on disease phenotypes and genetic profiles, to determine responders from non-responders, i.e., which individuals are most likely to benefit from these new treatment strategies. One systematic review investigated the potential of omics biomarkers in the field of RA, which identified 196 potential clinical biomarkers [48]; however, to the best of our knowledge, there are currently no well-established biomarkers or omics studies specifically tailored to optimizing tetracycline use in RA. This remains an area of ongoing research and presents a significant opportunity for future studies to enhance the precision and effectiveness of tetracycline therapies. Apart from biomarker identification, future studies should incorporate the exploration of comorbidities, such as cardiovascular disease and osteoporosis, which are often associated with RA, and assess the effects of tetracyclines on these conditions in combination. The longitudinal effects of tetracycline use should also be investigated in future studies.

Finally, the vast majority of available in silico methods should be employed to maximize the identification of tetracyclines’ potential before clinical trials. One example is drug repurposing approaches based on transcriptomic profiling, which can help select the most potent novel chemically modified tetracyclines based on the transcriptomic signature that the drug induces.

## 8. Conclusions and Perspectives

This comprehensive review highlights the importance of exploring novel treatment options for RA, a chronic autoimmune disease featured by persistent inflammation, joint pain, and stiffness, which increases the overall healthcare burden and diminishes the quality of life. Current treatment strategies primarily focus on controlling inflammation, relieving symptoms, and halting disease progression. However, in recent years, tetracyclines have been proposed as a supplementary treatment option. With their dual mode of action, both antimicrobial and immunomodulatory, the future of tetracyclines in RA treatment is a very promising field. Notably, tetracyclines are of particular interest due to their ability to suppress collagenase activity, which is a critical process in RA pathology. Despite this potential, their integration into clinical practice remains limited due to small-scale clinical trials with inconsistent evidence and concerns about antimicrobial resistance.

The synthesis of novel chemically modified tetracyclines could further enhance their therapeutic applicability while minimizing antibacterial effects. Strategies to overcome these challenges could also be in use of natural products and nanoformulations as a collaborative method to improve antimicrobial effectiveness while addressing issues such as limited bioavailability and the development of resistance, as demonstrated by recent progress in flavonoid-based nanoparticle formulations [49].

Nanomaterials, due to their high surface-to-volume ratio and adaptability, offer a cutting-edge platform for sustained antibacterial activity, and, when paired with natural compounds, they facilitate targeted delivery and enhanced therapeutic results [50].

These strategies highlight the promise of utilizing natural substances like flavonoids alongside advanced nanoformulations to optimize the delivery of tetracycline, reduce side effects, and tackle significant limitations, thereby opening avenues for successful clinical applications. However, further research is needed to support these potentials in natural compound and nanoformulation use [50,51].

In conclusion, tetracyclines hold promise as a supplementary option in RA management, but their current role remains largely experimental. Addressing existing evidence gaps, coupled with leveraging technological advancements and personalized medicine approaches, could enable tetracyclines to transition from a supplementary treatment option to unlocking their full potential in improving outcomes for patients with RA. Finally, for tetracyclines to be more fully integrated into the treatment of RA, close collaboration between rheumatologists, pharmacologists, molecular biologists, and bioinformaticians is needed to address current challenges, encourage creativity in drug development, and ultimately translate untried approaches into medical practice.

## Figures and Tables

**Table 1:** Summary of recent clinical evidence of tetracyclines in RA treatment.

Author, Year	Study Design	Antibiotics	Population	Conclusions
St Clair et al., 2001. [35]	RCT	Doxycycline vs. azithromycin vs. placebo	10 vs. 11 vs. 10	No evidence of reducing disease activity or collagen crosslink production
O’Dell et al., 2006. [36]	RCT	MTX and high-dose doxycycline vs. MTX and low-dose doxycycline vs. placebo with MTX	24 vs. 18 vs. 24	Initial therapy with MTX plus doxycycline was superior to treatment with MTX alone, not dependent on doxycycline dosage
Smith et al., 2011. [37]	Comparative study	Minocycline or doxycycline prescription	15,716 patients with RA	Minocycline or doxycycline as primary treatment for RA is not accepted and their use is primarily in patients with long-standing, refractory disease
Smith et al., 2011. [38]	RCT	Placebo vs. clindamycin with tetracycline	8 vs. 12	Halted before conclusion because it was unlikely that a significant difference in ACR50 response would emerge

Abbreviations: RCT—randomized controlled trial; MTX—methotrexate; RA—rheumatoid arthritis; and ACR50—American College of Rheumatology 50% improvement.

## Data Availability

Data sharing is not applicable to this article as no new data were created or analyzed in this study.
